# Four-Dimensional Transvaginal Ultrasonography as a First-Line Non-Invasive Follow-Up After Hysteroscopic Metroplasty in Dysmorphic Uterus Cases

**DOI:** 10.3390/jcm14217597

**Published:** 2025-10-26

**Authors:** Nurullah Peker, Abdurrahman Sengi, Elif Ucar, Talip Karacor, Ugur Deger, Elif Agacayak, Mehmet Sıddık Evsen

**Affiliations:** 1 Faculty of Medicine, Department of Gynecology and Obstetrics, Dicle University, 21280 Diyarbakır, Turkey; 2 Department of Gynecology and Obstetrics, Gazi Yaşargil Education and Research Hospital, 21070 Diyarbakır, Turkey; 3 Faculty of Health Sciences, Department of Midwifery, Istanbul Esenyurt University, 34510 Istanbul, Turkey; 4 Department of Obstetrics and Gynecology, Sakarya Education and Research Hospital, 54100 Sakarya, Turkey; 5 Faculty of Medicine, Department of Obstetrics and Gynecology, Uskudar University, 34768 Istanbul, Turkey

**Keywords:** dysmorphic uterus, hysteroscopic metroplasty, transvaginal ultrasonography, cost-effectiveness, reproductive outcomes

## Abstract

**Background/Objectives:** This study compared the cost-effectiveness of 4D transvaginal ultrasonography (TVUSG), hysterosalpingography (HSG), and hysteroscopy (HS) in evaluating postoperative uterine correction among patients who underwent hysteroscopic metroplasty for a dysmorphic uterus. Additionally, pregnancy outcomes following surgery were assessed. **Materials and Methods:** Thirty-one patients who underwent hysteroscopic metroplasty due to a dysmorphic uterus between July 2023 and June 2024 were retrospectively analysed. Postoperative uterine cavity evaluations were performed exclusively using 4D transvaginal ultrasonography (4D-TVUSG). The characteristics of alternative imaging modalities (HSG and HS) were evaluated conceptually in terms of invasiveness, feasibility, and cost based on official Ministry of Health data and previously published literature, rather than through direct patient-based comparison. One-year postoperative pregnancy outcomes were also recorded. **Results:** Notably, 4D-TVUSG was considered preferable to HSG and HS due to its non-invasive nature, ease of use, lack of anaesthesia requirement, and lower cost. Postoperatively, 14 patients (45.2%) achieved pregnancy, of whom 3 (21.4%) experienced miscarriages and 11 (78.6%) had ongoing pregnancies or live births. All patients demonstrated a normalised uterine cavity on follow-up and a 100% surgical success rate. **Conclusions:** It was found that 4D-TVUSG is a reliable, cost-effective, and non-invasive method for postoperative assessment in patients with a dysmorphic uterus undergoing hysteroscopic metroplasty. This is the first study to compare the cost-effectiveness of these three imaging methods, highlighting 4D-TVUSG as a potential first-line follow-up tool in clinical practice.

## 1. Introduction

A dysmorphic uterus, formerly known as a ‘T-shaped uterus’ in the American Fertility Society classification of anomalies of the Müllerian duct, is denoted as a second class (Class U1) uterine anomaly in the European Society of Human Reproduction and Embryology (ESHRE) and the European Society for Gynaecological Endoscopy (ESGE) consensus on the classification of congenital genital tract anomalies, and it leads to poor reproductive and obstetric outcomes [1,2].

The ESHRE and ESGE classification systems of female genital anomalies define a dysmorphic uterus as a narrow uterine cavity due to thickened lateral walls. This condition is characterised by a proportion of 2/3 uterine corpus and 1/3 cervix [1]. There is no consensus regarding the gold standard method for diagnosing a dysmorphic uterus. The best tool for examining the morphology of a uterine cavity is three-dimensional (3D) ultrasound, as neither hysterosalpingography (HSG) nor hysteroscopy (HS) provides information on uterine wall thickness or serosal contour [3]. Therefore, confirmation is typically performed using ultrasound-based imaging methods. The current consensus and recent reviews emphasise that three-dimensional ultrasonography is the preferred method for assessing uterine morphology and is widely used in clinical practice [2,3].

The prevalence of a T-shaped uterus and its impact on reproductive outcomes are unknown. The presence of uterine anomalies is not typically evaluated among asymptomatic women in the general population. Therefore, published studies on T-shaped uteruses have been conducted on patients with a history of adverse reproductive outcomes, such as subfertility, miscarriage, preterm birth or ectopic pregnancy [4,5]. In women with a T-shaped uterus, the altered shape of the uterine cavity and its reduced volume may contribute to diminished endometrial receptivity. These factors can impair uterine expansion during pregnancy, potentially resulting in poor reproductive and obstetric outcomes [6,7,8].

The prevalence of a T-shaped uterus in women with poor reproductive outcomes is 0.8% [6]. In symptomatic women, hysteroscopic metroplasty is considered a treatment option, but there is no robust evidence regarding its efficacy or safety [6]. Although studies have evaluated 3D ultrasonography, HS and HSG for the confirmation of anatomical correction after hysteroscopic metroplasty, there is no definitive opinion on which imaging technique should be used [6,8].

This study aims to present the reproductive results of patients who underwent hysteroscopic metroplasty for a dysmorphic uterus and to determine the best imaging method for confirming postoperative anatomic correction in these patients.

## 2. Materials and Methods

The hospital records of women who underwent hysteroscopic metroplasty due to a dysmorphic uterus between July 2023 and June 2024 were evaluated at the gynaecology and obstetrics clinics of a tertiary central university hospital. Before conducting this cohort study, approval was obtained from Dicle University Medical Faculty Ethics Committee for Non-Interventional Studies (date: 20 December 2023, approval no: 69). Patients with primary infertility or secondary infertility, a history of abortion and no living child who were diagnosed with a T- or Y-shaped uterus on HSG or 4D-TVUSG and underwent hysteroscopic metroplasty were included in the study. Exclusion criteria for this study included: mild and/or severe male infertility factors, history of endometriosis and/or endometrioma, tubal pathologies (e.g., bilateral tubal obstruction or hydrosalpinx), women with a history of tuberculosis and any prior uterine interventions (e.g., myomectomy, HS).

A T-shaped uterus was defined by HSG as follows: as a condensed lateral side uterine cavity observed using a properly performed HSG, and the angle of the narrowed side wall was almost 90°, in which the traction to the uterus was sufficient to expose the normal plane of the uterine cavity. In addition to the previously established criteria, the diagnosis of a Y-shaped uterus on an HSG was made by identifying a condensed area at the fundus, like that seen on lateral uterine walls. Patients who underwent HSG and were diagnosed with a T- or Y-shaped uterus were confirmed with 3D ultrasonography. For the diagnosis of a T-shaped uterus with 3D ultrasonography, criteria defined by the Congenital Uterine Malformation by Experts were used [9].

At the proliferative phase of the menstrual cycle (days 3–7 after the menses phase), operative HS was performed in the lithotomy position under sedation anaesthesia with a proper dosage of propofol (1–2 mg/kg) and midazolam (0.02–0.04 mg/kg). The cervix was dilated to 4.5–9 mm with Hegar bougies, and the uterine depth was measured with a hysterometer. An 8.5 mm Hystero-Resectoscope system (Olympus) was placed into the cavity. After exhausting air, a 0.9% NaCl solution was applied as a distention medium with an irrigation pressure of 120 mmHg. The scope successively entered the ectocervix, endocervical canal and endocervix.

The uterine cavity, both tubal ostia, the condition of the endometrium and the ratio between endocervical canal length and uterine cavity depth were examined to rule out other lesions. After evaluation of the uterine cavity, the lateral side walls were cut longitudinally using a bipolar needle tip resectoscope until the tube orifice was visible from the isthmus.

Operations were performed by three experienced surgeons. At the end of the operation, no anti-adhesive gel and/or intrauterine balloon was used. After an operation, the patient was discharged on the same day. The patients were prescribed 2 mg of estradiol valerate oral oestrogen support for two menstrual cycles (Cyclo-Progynova tb, Bayer). All patients were counselled to prevent them from conceiving until the second evaluation with 4D vaginal ultrasonography. After two menstrual cycles, 4D vaginal ultrasonography was repeated to evaluate the newly formed uterine cavity.

After surgery, the uterine cavity was evaluated with 4D vaginal ultrasonography to confirm normalisation. Once the cavity was determined to be normal, patients were allowed to pursue pregnancy through natural conception, intrauterine insemination or IVF. Pregnancy outcomes were then recorded following a one-year follow-up period. Surgical success was defined as the achievement of a normal uterine cavity by 4D vaginal ultrasonography performed after two cycles of postoperative medical treatment. The reproductive outcomes were recorded using the delivery registry from our hospital database and telephone interviews.

When interviewed by telephone, subjects were informed about the purpose of this study, and they could have refused to participate without giving a reason. Clinical pregnancy was defined as amenorrhea, a positive blood human chorionic gonadotropin test, and diagnosed via ultrasonographic visualisation of one or more gestational sacs in the intrauterine cavity. An abortion was defined as the spontaneous loss of a clinical pregnancy before 20 weeks of completed gestation. An ongoing pregnancy was defined as an ultrasound-confirmed viable intrauterine pregnancy that had reached at least 20 weeks’ duration.

The patients were divided into two groups: T-shaped uterus and Y-shaped uterus. Parameters such as age, gravidity, parity, abortion, number of living children, infertility type, history of assisted reproductive treatment before the procedure, history of previous surgery, hospital admission complaint, pregnancy status after metroplasty, outcome of pregnancy and conception method were compared between the two groups. At the same time, the prices determined by the ministries of health in different countries for imaging methods such as 4D vaginal ultrasonography, HSG and HS to detect uterine recovery in the postoperative period were compared.

### Statistical Analysis

The IBM Statistical Package for the Social Sciences Version 21.0 for Windows (IBM Corp.: Armonk, NY, USA) was used to analyse data. The normal distribution of the dataset was confirmed by a Kolmogorov–Smirnov test, and data were presented as mean ± standard deviation (mean ± SD) and median (min–max). Categorical variables were presented as numbers (n) and percentages (%). Group comparisons of categorical variables were made using a Fisher’s Exact Test and a Fisher–Freeman–Halton Exact Test, and continuous variables were made using an Independent-Samples Mann–Whitney U Test and Yates’s chi-squared test. The results were evaluated with a significance level of *p* < 0.05.

## 3. Results

During the study period, 31 patients who met the inclusion criteria and underwent hysteroscopic metroplasty due to a dysmorphic uterus were evaluated. Surgical success was determined to be 100%. Fifteen patients underwent hysteroscopic metroplasty for a T-shaped uterus and 16 patients for a Y-shaped uterus.

A comparison of demographic and clinical data between the two groups is given in Table 1. Compared to invasive methods, such as HSG and HS, in determining the correction in the uterine cavity in the postoperative period, 4D vaginal ultrasonography was found to be a less expensive examination, as it is non-invasive and easier to access and apply. The Ministry of Health data of different countries [10,11,12,13,14,15] regarding the charges for 4D vaginal ultrasonography, HSG and HS that were applied to determine the correction in the postoperative uterine cavity are summarised in Table 2.

## 4. Discussion

In patients who underwent hysteroscopic metroplasty due to a dysmorphic uterus, 3D vaginal ultrasonography, HSG and HS are used to determine the normalisation in the uterine cavity in the postoperative period. It has been observed that HSG and HS are not the first choice because they are invasive methods, require anaesthesia and are expensive and not easily accessible compared to vaginal ultrasonography. In this study, it was determined that the best method for determining postoperative normalisation in the uterine cavity in patients who underwent hysteroscopic metroplasty was 4D-TVUSG because it is a non-invasive method, does not require anaesthesia, is inexpensive and is easily accessible. These results are consistent with recent comprehensive reviews highlighting the effectiveness of 2D, 3D, and particularly 4D ultrasonography for the accurate evaluation of uterine morphology and anomalies [16].

Invasive methods, such as HSG and HS, which are commonly used for postoperative evaluation, may cause physical discomfort during the procedure, require anaesthesia, and impose additional financial burdens on hospitals and healthcare systems. Moreover, these methods are associated with various complications. The reported adverse effects of HSG include uterine cramping, infection, vaginal bleeding, peritonitis and, although rare, intraperitoneal contrast leakage. Studies have shown that approximately 85% of women undergoing HSG experience pain, with nearly half reporting moderate to severe intensity [17,18]. HSG is frequently experienced as painful and anxiety-inducing despite various mitigation efforts, which makes the practicality of a non-invasive, anaesthesia-free follow-up approach even more preferable. [19]. The overall complication rate in operative hysteroscopy procedures is approximately 1–3%, with reported incidences including uterine perforation (0.1–2%), cervical laceration (1–11%), infection (0.7–1%), and, although rare, venous gas embolism.

These findings highlight that, due to its invasive nature, HS carries a risk of various complications [20,21,22]. These complications compromise both patient comfort and procedural safety. In contrast, 4D-TVUSG is a completely non-invasive method that carries no risk of procedure-related physical trauma or infection. This characteristic makes 4D-TVUSG particularly advantageous for patients requiring repeated evaluations. Furthermore, since it causes significantly less stress for the patient and does not require cervical manipulation during the procedure, the risk of complications is virtually negligible. Therefore, in terms of both patient safety and procedural comfort, 4D-TVUSG offers a clear advantage over invasive methods for postoperative assessment.

HS is most often performed under sedation or general anaesthesia. This requirement prolongs pre-procedural patient preparation, increases overall costs, and introduces the risk of anaesthesia-related complications [23]. Particularly in low- and middle-income countries, the use of sedation is limited due to additional costs, which may negatively impact patient comfort [24]. Complications that may occur during procedures performed under sedation or general anaesthesia include respiratory depression, hypotension, drug reactions, nausea and vomiting, dizziness and, rarely, cardiac arrhythmias. In the literature, the incidence of sedation-related complications is reported to be approximately 1–2%, with higher rates observed in elderly, obese or comorbid patients [25].

Although HSG is generally a procedure that does not require anaesthesia, sedation support may be necessary in certain cases due to severe pain anticipation or past traumatic experiences. Moreover, vasovagal reactions caused by pain related to contrast agent administration or cervical manipulation are not uncommon [26]. However, 4D-TVUSG does not require anaesthesia under any circumstances. This provides a significant comfort advantage for the patient and eliminates the risk of systemic complications associated with anaesthesia. Especially for patient groups requiring low-risk, rapid and safe evaluation, 4D-TVUSG is a highly preferable option.

The use of invasive methods such as HSG and HS involves equipment, contrast agents, sterilisation requirements and the need for anaesthesia, all of which contribute to the overall cost of these procedures. Additional factors, such as post-procedural observation time, treatment of potential complications and healthcare personnel labour, further increase the total expense. In a comprehensive analysis published by Ridley in 2011 [27], TVUSG was shown to provide higher diagnostic accuracy and significantly lower costs compared to HSG in detecting uterine abnormalities. Furthermore, the recent literature has emphasised that three-dimensional (3D) ultrasonography offers high diagnostic accuracy and remains a widely used tool in the evaluation of dysmorphic uterus. Importantly, four-dimensional (4D) ultrasonography demonstrates comparable diagnostic performance to 3D, while providing the additional advantage of real-time volumetric assessment, which may further enhance clinical decision-making in postoperative follow-up [2]. Although 3D and 4D ultrasonography are based on the same volumetric imaging principles and provide comparable diagnostic accuracy, the main advantage of 4D ultrasonography lies in its ability to visualise volumetric data in real time and dynamically. This feature is particularly valuable in the postoperative follow-up of patients undergoing hysteroscopic metroplasty, as it allows a more precise assessment of uterine cavity remodelling and the healing process over time. Furthermore, in this study, 4D ultrasonography was observed to improve workflow efficiency and enhance patient comfort without compromising diagnostic performance. These advantages suggest that 4D ultrasonography is a practical and effective alternative, especially in clinical follow-up settings where repeated evaluations are required.

According to Medicare reimbursement rates cited in the study, the cost of TVUSG was approximately USD 138, while HSG cost USD 409, and hysteroscopy ranged between USD 228 and USD 542. Furthermore, due to the need for additional imaging techniques (e.g., MRI, a second HS) for diagnostic confirmation, the total cost of an HSG-based diagnostic pathway may reach between USD 3800 and USD 11,800. In contrast, the need for such additional imaging is considerably lower when the initial diagnosis is made using TVUSG. In light of these findings, 4D-TVUSG appears to be a more economical, non-invasive, and highly accurate option for evaluating the uterine cavity, particularly in a postoperative setting. In our study, an examination of data obtained from the healthcare systems of various countries revealed that the costs of HSG and HS procedures are considerably high.

Compared to other imaging modalities, TVUSG is not only more economical in terms of equipment but also requires less time and fewer personnel resources. Its repeatability and feasibility in outpatient settings further enhance its economic advantages. Particularly in healthcare systems with limited resources or in situations where patients are cost sensitive, 4D-TVUSG should be prioritised as the first-line imaging method. From this perspective, 4D-TVUSG stands out for its medical effectiveness and cost-effectiveness. Due to its ease of application, minimal burden on patients and economic advantages for healthcare systems, it represents an ideal option for postoperative follow-up after dysmorphic uterus surgery.

Hysteroscopic metroplasty significantly improves pregnancy rates, particularly in women diagnosed with a T-shaped or dysmorphic uterus who have a history of infertility or recurrent pregnancy loss. For instance, in a retrospective 111-person cohort study conducted in China, the pregnancy rate in the congenital anomaly group increased from 28.3% to 87.0%, and the live birth rate rose from 23.1% to 79.5% following metroplasty [28]. In large-sample studies, pregnancy rates have been reported to range between 49.5% and 66.1%, while live birth rates vary between 63.2% and 86.7%. In these studies, miscarriage rates ranged from 11.9% to 28.1% [29,30,31].

However, within the scope of a comprehensive systematic review and meta-analysis, the overall rate of clinical pregnancy following metroplasty was reported as 68.9%, while the live birth rate was 56.2% [32]. In another prospective study following metroplasty in 83 women, the clinical pregnancy rate was reported as 77.1%, the live birth rate as 79.7% and the abortion rate as only 9.4%; no serious pregnancy-related complications associated with the procedure were observed [33].

In patients with infertility and multiple IVF failures, the live birth rate following metroplasty was reported as 27.9% at 18 months of follow-up and 53.5% over a longer period; notably, the spontaneous pregnancy rate was 28.6% [34]. In our study, during the one-year follow-up of 31 patients who underwent hysteroscopic metroplasty, 14 women (45.2%) were found to have conceived. Among those who became pregnant, 3 (21.4%) experienced miscarriages, while 11 (78.6%) had ongoing pregnancies or delivered successfully. Of the women who conceived, nine (64.3%) had a Y-shaped uterus and five (35.7%) had a T-shaped uterus. These findings suggest that anatomical correction through surgery in cases of a dysmorphic uterus may increase pregnancy rates and improve the likelihood of a live birth [35].

A major strength of this study is that, unlike most previous research that focused on invasive methods, such as HSG or office hysteroscopy, to evaluate anatomical correction after metroplasty, this study demonstrated that 4D-TVUSG—a non-invasive, cost-effective and practical method—can serve as a reliable alternative. To our knowledge, this is the first study to compare three different imaging modalities in terms of cost-effectiveness following dysmorphic uterus surgery, making it a valuable contribution to the literature.

However, this study has limitations, including a relatively small sample size, which is due to the rarity of dysmorphic uterus and the single-centre nature of the study, as well as the absence of long-term pregnancy and birth outcome data. In addition, postoperative evaluation was performed exclusively with 4D transvaginal ultrasonography; therefore, a direct diagnostic comparison with HSG or hysteroscopy could not be conducted. The comparison among the imaging methods was conceptual, based on literature data and official cost analyses rather than patient-based findings.

## 5. Conclusions

This study demonstrated that 4D-TVUSG is a reliable, accessible and cost-effective method for evaluating postoperative anatomical correction in patients who underwent hysteroscopic metroplasty due to a dysmorphic uterus. While follow-up with HSG and HS may not always be feasible in resource-limited settings, detailed assessment of a uterine cavity is achievable with 4D-TVUSG. These findings support the use of 4D-TVUSG as a diagnostic tool as an effective method for postoperative monitoring.

## Figures and Tables

**Table 1:** Comparison of clinical and demographic data between the two groups who underwent hysteroscopic metroplasty.

Characteristics		T-Shaped n = 15	Y-Shaped n = 16	*p*
Age mean ± SD—median (min–max)		28.60 ± 3.72—29(22–36)	33.44 ± 6.60—32(23–44)	0.033 ^a^
Gravidity mean ± SD—median (min–max)		0.53 ± 0.74—0(0–2)	1.88 ± 1.41—2(0–5)	0.004 ^a^
Parity mean ± SD—median (min–max)		0—0	0.63 ± 0.96—0(0–3)	0.078 ^a^
Abortion mean ± SD—median (min–max)		0.53 ± 0.74—0(0–2)	1.25 ± 1.13—1(0–3)	0.078 ^a^
Number of living children mean ± SD—median (min–max)		0—0	0.38 ± 0.89—0(0–3)	0.379 ^a^
Infertility type n (%)	Primary	9(75)	3(25)	0.029 ^b^
	Secondary	6(31.6)	13(68.4)	
History of assisted reproductive treatment before the procedure n (%)	No treatment	8(40)	12(60)	0.097 ^c^
	Failed IUI	5(71.4)	2(28.6)	
	Failed IVF	0(0)	2(100)	
	Failed IUI and IVF	2(100)	0(0)	
History of previous surgery n (%)	No	12(46.2)	14(53.8)	0.689 ^c^
	HS	2(66.7)	1(33.3)	
	LS	1(100)	0(0)	
	LS + HS	0(0)	1(100)	
Hospital admission complaint n (%)	Infertility	9(75)	3(25)	0.029 ^b^
	Abortion	6(31.6)	13(68.4)	
Pregnancy status after metroplasty n (%)	Yes	5(35.7)	9(64.3)	0.357 ^d^
	No	10(58.8)	7(41.2)	
Outcome of pregnancy n (%)	Not pregnant	10(58.8)	7(41.2)	0.494 ^c^
	Abortion	1(33.3)	2(66.7)	
	Ongoing pregnancy	4(36.4)	7(63.6)	
Method of conception n (%)	Not pregnant	10(58.8)	7(41.2)	0.564 ^c^
	Spontaneous	3(33.3)	6(66.7)	
	IUI	0(0)	1(100)	
	İVF	2(50)	2(50)	

^a^ Independent-Samples Mann–Whitney U Test ^b^ Fisher’s Exact Test ^c^ Fisher–Freeman–Halton Exact Test ^d^ Yates chi-squared test.

**Table 2:** Ministry of Health data of different countries regarding the charges for TVUSG, HSG and HS.

Country	TVUSG	HSG		HS	
		Without anaesthesia	With anaesthesia	Without anaesthesia	With anaesthesia
Turkey [10,11]	2.1–3 €	3.6–5.1 €	4.7–7 €	29.2- 48.7 €	30.4–49.9 €
Germany [12]	25–53 €	220–350 €	700–900 €	170–200 €	420–450 €
Italy [13,14]	59.5–87.5 €	125 €	200–400 €	200 €	300–500 €
United States of America [15]	125–175 $	400–1800 $	600–2000 $	360–550 $	1300–1500 $

TVUSG: transvaginal ultrasonography, HSG: hysterosalpingography, HS: hysteroscopy €: Euro $: Dollar.

## Data Availability

The original contributions presented in this study are included in the article. Further inquiries can be directed to the corresponding author(s).
